# Enzyme-Assisted Extraction of Bioactive Material from *Chondrus crispus* and *Codium fragile* and Its Effect on *Herpes simplex* Virus (HSV-1)

**DOI:** 10.3390/md13010558

**Published:** 2015-01-16

**Authors:** Garima Kulshreshtha, Anne-Sophie Burlot, Christel Marty, Alan Critchley, Jeff Hafting, Gilles Bedoux, Nathalie Bourgougnon, Balakrishnan Prithiviraj

**Affiliations:** 1 Department of Environmental Sciences, Faculty of Agriculture, Dalhousie University, PO Box 550, Truro, NS B2N 5E3, Canada; E-Mail: gr784654@dal.ca; 2 Laboratoire de Biotechnologie et Chimie Marines, EA3884, UBS, IUEM, F-56000 Vannes, France; E-Mails: anne-sophie.burlot@univ-ubs.fr (A.-S.B.); Christel.Marty@univ-ubs.fr (C.M.); gilles.bedoux@univubs.fr (G.B.); nathalie.bourgougnon@univ-ubs.fr (N.B.); 3 Acadian Seaplants Limited, 30 Brown Avenue, Dartmouth, NS B3B 1X8, Canada; E-Mails: Alan.Critchley@acadian.ca (A.C.); jhafting@acadian.ca (J.H.)

**Keywords:** *Codium fragile*, *Chondrus crispus*, red seaweeds, sulfates, *Herpes simplex* virus (HSV-1)

## Abstract

*Codium fragile* and *Chondrus crispus* are, respectively, green and red seaweeds which are abundant along the North Atlantic coasts. We investigated the chemical composition and antiviral activity of enzymatic extracts of *C. fragile* (CF) and *C. crispus* (CC). On a dry weight basis, CF consisted of 11% protein, 31% neutral sugars, 0.8% sulfate, 0.6% uronic acids, and 49% ash, while CC contained 27% protein, 28% neutral sugars, 17% sulfate, 1.8% uronic acids, and 25% ash. Enzyme-assisted hydrolysis improved the extraction efficiency of bioactive materials. Commercial proteases and carbohydrases significantly improved (*p* ≤ 0.001) biomass yield (40%–70% dry matter) as compared to aqueous extraction (20%–25% dry matter). Moreover, enzymatic hydrolysis enhanced the recovery of protein, neutral sugars, uronic acids, and sulfates. The enzymatic hydrolysates exhibited significant activity against *Herpes simplex* virus (HSV-1) with EC_50_ of 77.6–126.8 μg/mL for CC and 36.5–41.3 μg/mL for CF, at a multiplicity of infection (MOI) of 0.001 ID_50_/cells without cytotoxity (1–200 μg/mL). The extracts obtained from proteases (P1) and carbohydrases (C3) were also effective at higher virus MOI of 0.01 ID_50_/cells without cytotoxity. Taken together, these results indicate the potential application of enzymatic hydrolysates of *C. fragile* and *C. crispus* in functional food and antiviral drug discovery.

## 1. Introduction

Viruses cause life-threatening diseases such as hepatitis, influenza, HIV-AIDS, ebola, and herpes. Hence, there is a concerted effort to discover antiviral agents to control infection and the spread of infectious diseases. The *Herpes simplex* virus (HSV) is a double-stranded DNA-enveloped virus, and a common human pathogen. There are two strains: HSV-Type 1 (HSV-1) and HSV-Type 2 (HSV-2). HSV-1 is frequently associated with oral and facial infections and encephalitis, whereas HSV-2 causes genital tract infections [1]. In the United States, 60%–85% of adults, by the age of 60 years, have HSV-1 antibodies, and 6%–50% at the age of 40 have HSV-2 antibodies in their blood [2]. HSV enters the host through mucous membranes or skin lesions into the orolabial epithelial cells. This entry is mediated by viral envelope proteins, such as viral glycoproteins gB, gC, gD, gH, and gL. HSV adsorption by target cells involves the interaction between cell-surface heparin sulfate (HS) and viral glycoprotein. Upon successful adsorption, the virus replicates by rapid cell-to-cell multiplication within the epithelial cells. Further, the virus is transported by infected sensory neuritis to trigeminal ganglia, where it establishes long-term latency within infected sensory neurons [3,4,5]. Recurrent infections with HSV are more frequent in people with acquired immune deficiency (AIDS) and can lead to high mortality. HSV infection is one of the first opportunistic infections in AIDS patients. Therefore, its control is considered to be a primary approach in the clinical management of HIV [6,7,8]. Similarly, primary or recurrent HSV in the genital tract of a mother can lead to neonatal HSV infections. In the United States, incidences of neonatal HSV are common (8 to 60 per 100,000). A newborn child with HSV infection is predisposed to acute meningoencephalitis, neurological disorders, skin and eye lesions, and disseminated diseases [9,10]. HSV-associated diseases are also recurrent in allogeneic bone marrow patients (70%–80%) and solid organ transplant patients (32%–53%) [11,12].

The common anti-HSV drugs include acyclic nucleosides Acyclovir (ACV). Acyclovir is a guanosine analog that interferes with viral DNA synthesis and prevents chain elongation and viral replication [13]. Recently, other nucleoside analogs, such as Penciclovir, and Ganciclovir and their derivatives valacyclovir and famciclovir, all of which interfere with viral DNA replication, have been introduced for human anti-HSV treatments [14,15]. Although acyclovir and its derivatives are very effective antiherpes drugs, there is an emergence of drug-resistant mutants due to prolonged use of this drug [16]. Cidofovir, which is commonly used for the treatment of acyclovir- and foscarnet-resistant HSV infections [14], is nephrotoxic and therefore its use is restricted to patients with normal renal functions. Therefore, there is an immediate need for the development of novel antiherpetic agents [8]. Antiviral chemotherapy currently faces several obstacles including: toxicity of therapeutic molecules, interference with normal cellular metabolism, genetic variability (source of antiviral resistant mutants), and the incurable nature of latent infections.

More than 70% of the world’s surface is covered with oceans, teeming with marine organisms containing diverse bioactive compounds. Marine macroalgae, commonly referred to as seaweeds, are good sources of food and raw materials for cosmetic and the pharmaceuticals industry, and also have agricultural uses [17]. Seaweeds are rich in novel bioactive polysaccharides showing antimicrobial, antioxidant and antiviral activities. The edible red macroalga (Rhodophyta) *Chondrus crispus* is abundant along parts of the North Atlantic coast, where it is collected for carrageenan extraction [18]. Selected strains are also commercially cultivated on-land by Acadian Seaplants Limited in Nova Scotia [19,20]. Carrageenans and galactans are the main matrix polysaccharides of red seaweeds. These polysaccharides are formed by alternating units of 3-linked β-d-galactopyranosyl and 4-linked α-d/l-galactopyranosyl and have been shown to block viral replication and adsorption to the host cell surface [21,22,23,24,25]. *Codium fragile*, is an invasive green alga in Nova Scotia. It is now widely distributed along the east coast of North America [26], as well as in Europe. In Japan, *C. fragile* is used as food, and it is also used to treat diseases such as enterobiasis, dropsy, and dysuria, as documented in Asian medical textbooks [8]. Sulfated polysaccharides, such as glucuronic acid, iduronic acid, and rhamnan sulfates isolated from green seaweeds, including *Ulva*, *Enteromorpha*, and *Monostroma attisimum*, have been reported previously to have antiviral activities [13]. Therefore, seaweed-derived polysaccharides can be potential sources of functional bioactives, effective against viruses. Generally, the bioactive components of seaweeds are extracted using solvents and strong alkali. In recent years, enzyme-assisted extraction has gained attention as an effective tool to improve the extraction yield of bioactive compounds from seaweeds [27]. Additionally, this extraction method has been reported to increase the bioavailability of antiviral compounds such as polysaccharides, diterpenes, or glycolipids present in the seaweeds. Moreover, enzyme-assisted extraction is a solvent-free, eco-friendly, and cost-effective extraction method [2,28].

The aim of this study was to examine the use of three commercial carbohydrases and proteases to improve the extraction efficiency of bioactive materials from the selected red and green seaweeds, *C. crispus* (multiaxial filamentous tissue) and *C. fragile* (a coenocytic tissue), respectively. The recovery of chemical compounds using different enzymes was analyzed and then tested for antiviral activity against HSV-1 by a cytotoxic assay based on cell viability.

## 2. Results

### 2.1. Composition of Acid Hydrolysis Extract

The chemical composition of *Chondrus crispus* (CC) was significantly different from that of *Codium fragile* (CF) (Table 1). Dried CC contained 27.2% ± 1.4% protein, 17.6% ± 0.1% sulfates, and 1.8% ± 0.07% uronic acid. The dried CF samples contained 11.7% ± 0.3% proteins, 0.8% ± 0.1% sulfates, and 0.6% ± 0.01%uronic acid. The ash and neutral sugar content of the extract of CF was significantly (*p* ≤ 0.0001, *n* = 9) higher (49.9% ± 0.2%, 31.1% ± 0.4%) than that of CC (25% ± 0.3%, 28.8% ± 0.5%). CC had higher (*p* ≤ 0.0001, *n* = 9) proteins, sulfates, and uronic acids. The protein, sugars, sulfates, and uronic acid contents of 2 h hydrolysate were higher than those of 5 h hydrolysates (data not shown). 

**Table 1:** Chemical composition of *Chondrus crispus* (CC) and *Codium fragile* (CF) (raw matter after 2 h of hydrolysis) ^1^.

Algae (Freeze-Dried) ^2^	Proteins	Neutral Sugars	Sulfate Groups	Uronic Acids	Ash
*Chondrus crispus*	27.2 ± 1.4 ^a^	28.8 ± 0.5 ^a^	17.6 ± 0.1 ^a^	1.8 ± 0.07 ^a^	25 ± 0.3 ^b^
*Codium fragile*	11.7 ± 0.3 ^b^	31.1 ± 0.4 ^b^	0.8 ± 0.1 ^b^	0.6 ± 0.01 ^b^	49.9 ± 0.2 ^a^
*P-value*	≤0.0001	0.028	≤0.0001	≤0.0001	≤0.0001

^1^ In percentage dry matter hydrolysate (1 mg/mL); ^2^ Values are means ± SD (standard deviation) for each chemical analysis (nine replicates per treatment). Values with different superscript letters are significantly different (*p* < 0.05).

### 2.2. Biomass Yield

**Figure 1:**
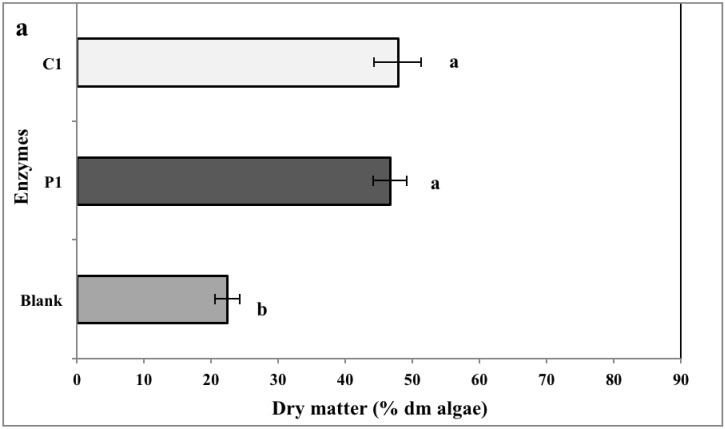

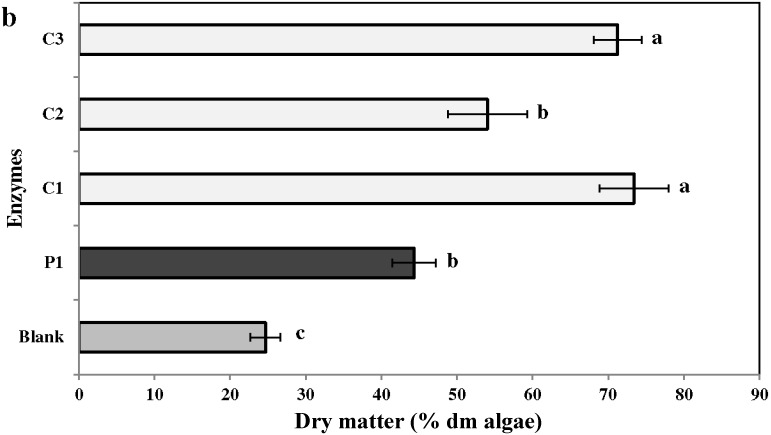
The repartitioning rate of algal dry matter (percentage dry matter yield) obtained with enzymatic hydrolysis of (**a**) *Codium fragile* (CF) and (**b**) *Chondrus crispus* (CC). Values with different superscript letters are significantly different (*p* < 0.05). Values represent mean ± standard deviation from three independent experiments (*n* = 9). P1: protease 1; C1: carbohydrases 1; C2: carbohydrases 2; C3: carbohydrases 3.

The repartitioning rate of algal dry matter is shown in Figure 1. Enzymatic extraction significantly increased the dry matter yield. In CF, the extraction yields with carbohydrases (C1) and proteases (P1) was 47.8% ± 3.5% and 46.6% ± 2.5%, respectively, which were higher (*p* ≤ 0.001) than control (water blank, 22.4% ± 1.9%). With CC*,* the higher percentage (*p* ≤ 0.001) of dry matter was obtained in supernatants treated with carbohydrases C1 and C3 (73.4% ± 3.3% and 71.2% ± 7.7%, respectively). Water extraction (water blank), under similar conditions of temperature and duration, showed lower dry matter yield (CF, 22.4% ± 1.9% and CC, 24.7% ± 2.1%, respectively). This indicated that the use of enzymes increased the extraction efficiency, in comparison to a water extraction. 

### 2.3. Chemical Analysis of Enzymatic Hydrolysates

**Figure 2:**
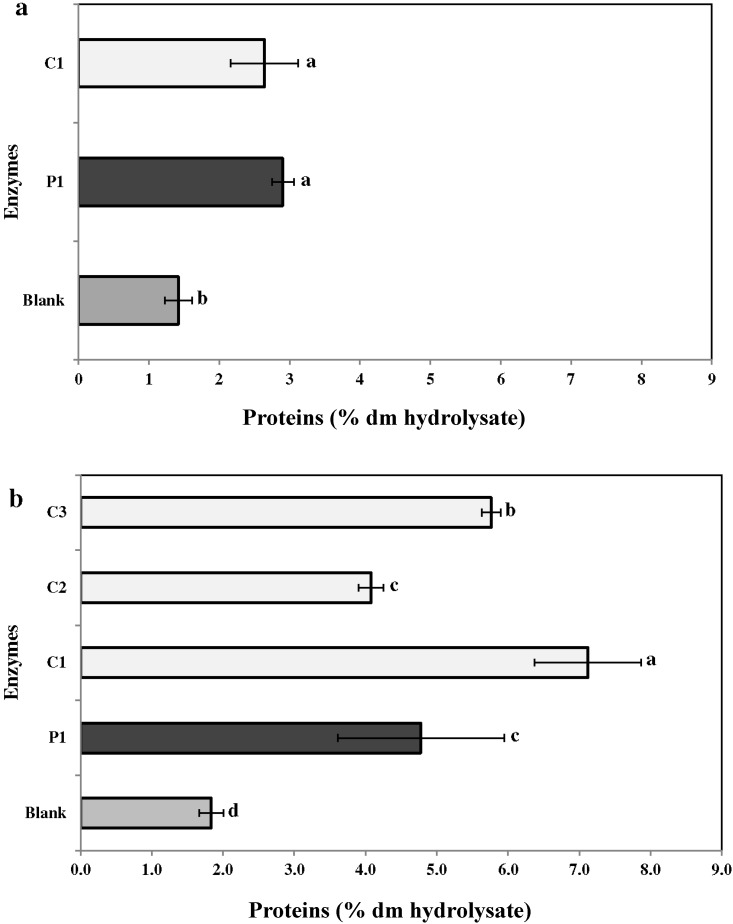
Total protein content in enzymatic hydrolysates of (**a**) *Codium fragile* (CF) and (**b**) *Chondrus crispus* (CC). Values with different superscript letters are significantly different (*p* < 0.05). Values represent mean ± standard deviation from three independent experiments (*n* = 9). P1: protease 1; C1: carbohydrases 1; C2: carbohydrases 2; C3: carbohydrases 3.

**Figure 3:**
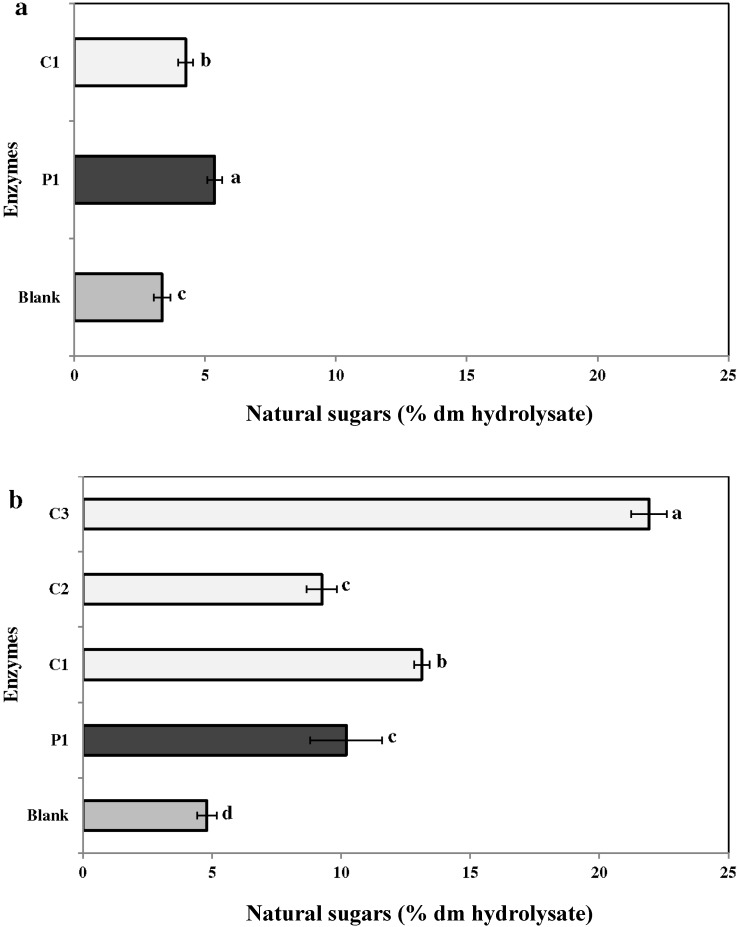
Content of neutral sugars in enzymatic hydrolysates from the two seaweeds (**a**) *Codium fragile* (CF) and (**b**) *Chondrus crispus* (CC). Values with different superscript letters are significantly different (*p* < 0.05). Values represent mean ± standard deviation from three independent experiments (*n* = 9). P1: protease 1; C1: carbohydrases 1; C2: carbohydrases 2; C3: carbohydrases 3.

**Figure 4:**
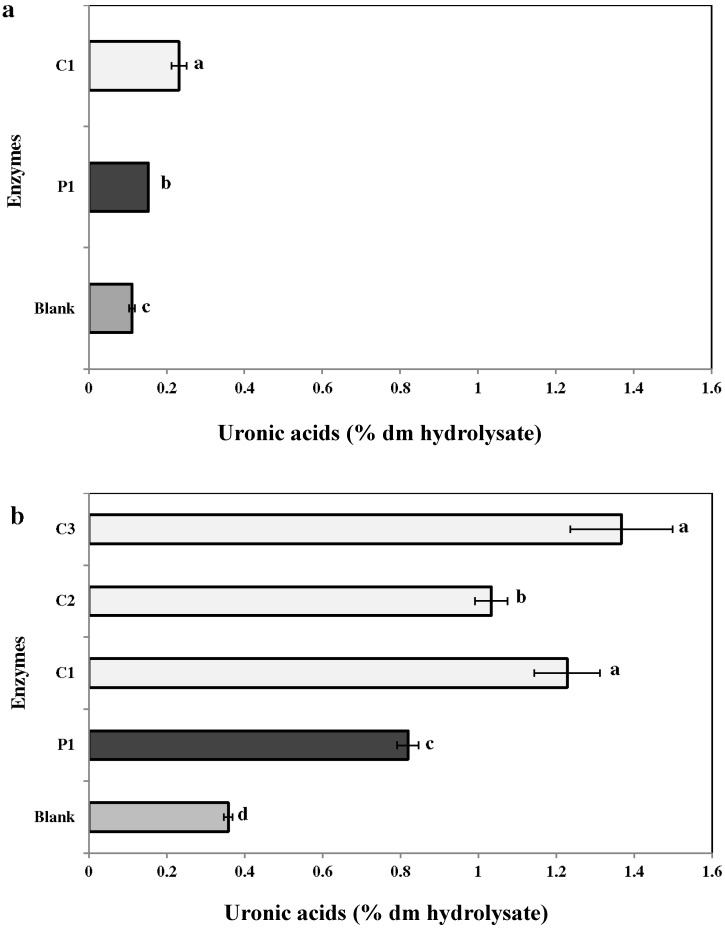
Uronic acids in enzymatic hydrolysates of (**a**) *Codium fragile* (CF) and (**b**) *Chondrus crispus* (CC). Values with different superscript letters are significantly different (*p* < 0.05). Values represent mean ± standard deviation from three independent experiments (*n* = 9). P1: protease 1; C1: carbohydrases 1; C2: carbohydrases 2; C3: carbohydrases 3.

**Figure 5:**
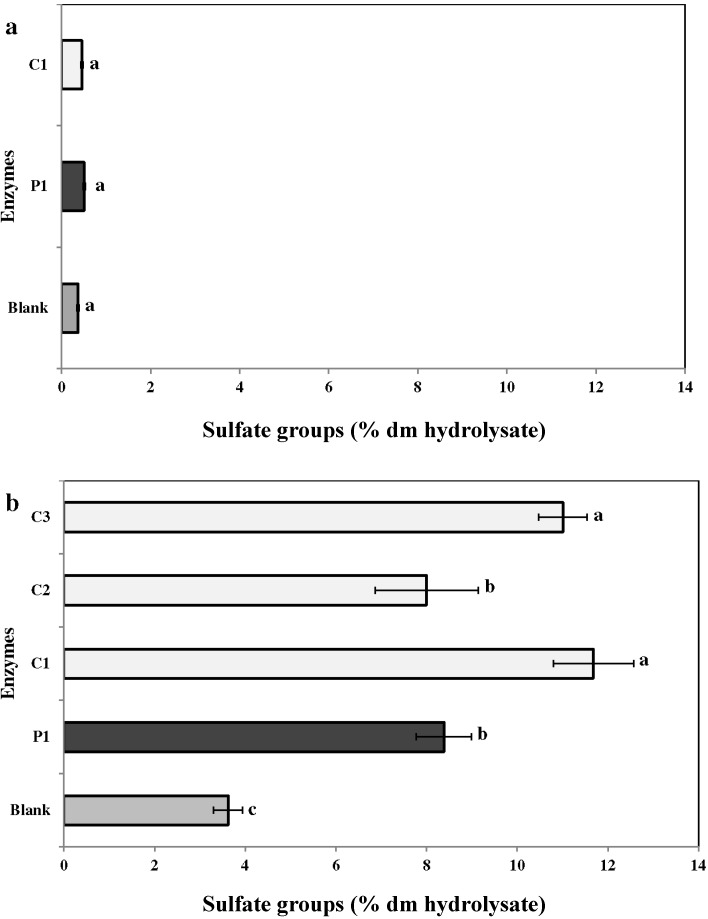
Sulfate groups in the enzymatic hydrolysates of (**a**) *Codium fragile* (CF) and (**b**) *Chondrus crispus* (CC). Values with different superscript letters are significantly different (*p* < 0.05). Values represent mean ± standard deviation from three independent experiments (*n* = 9). P1: protease 1; C1: carbohydrases 1; C2: carbohydrases 2; C3: carbohydrases 3.

**Figure 6:**
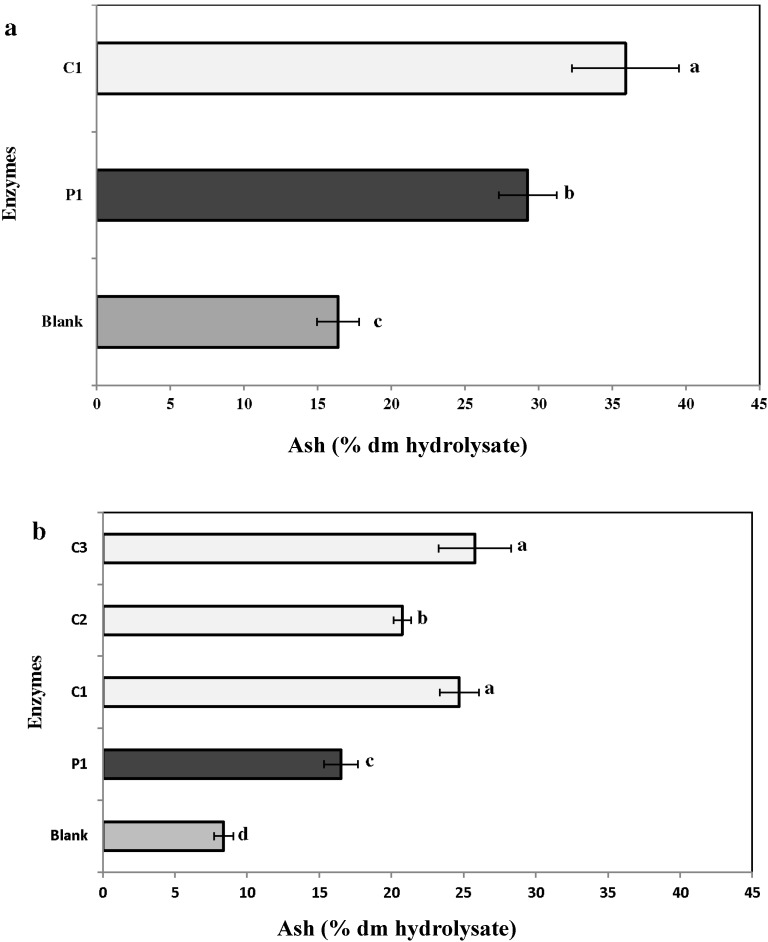
Total ash content in the enzymatic hydrolysates of (**a**) *Codium fragile* (CF) and (**b**) *Chondrus crispus* (CF). Values with different superscript letters are significantly different (*p* < 0.05). Values represent mean ± standard deviation from three independent experiments (*n* = 9). P1: protease 1; C1: carbohydrases 1; C2: carbohydrases 2; C3: carbohydrases 3.

The recovery of chemical fractions from the enzymatic hydrolysis of the two seaweeds is shown in Figure 2, Figure 3, Figure 4, Figure 5 and Figure 6. The percentage yield of each chemical compound in the hydrolysates is based on their dry weight percentage, obtained from the initial algal sample. Of all the chemical components, carbohydrases exhibited the highest percentage of dry matter in the hydrolysates.

#### 2.3.1. Proteins

Recovery of protein in the enzymatic extract of *C. crispus* was higher than that for *C. fragile* (Figure 2a,b). In *C. crispus,* the percentage recovery was highest with C1 (7.1% ± 0.3%) and ranged between 4.1% ± 0.4% and 5.8% ± 0.5% for other enzymes; this was significantly higher (*p* ≤ 0.05) than the control (water, 1.8% ± 0.1%). Similarly, with *C. fragile*, the percentage yield with enzymes (P1: 2.9% ± 0.1% and C1: 2.6% ± 0.2%) was higher than that of the control (1.4% ± 0.1%) (Figure 2b). Overall, the three carbohydrases were the most effective enzymes for the protein recovery (Figure 2a,b).

#### 2.3.2. Neutral Sugars

Enzymatic extraction was an effective method to improve recovery of neutral sugars (Figure 3a,b). The percentage of neutral sugar in the enzymatic hydrolysates ranged from 9.3% ± 0.2% (C2) to 21.9% ± 0.4% (C3) in *C. crispus* and 4.3% ± 0.2% (C1) to 5.4% ± 0.3% (P1) in *C. fragile*. Overall, the neutral sugars content extracted with carbohydrases was the highest.

#### 2.3.3. Uronic Acid

The concentration of uronic acid was significantly higher (*p* ≤ 0.05) in the enzymatic extracts as compared to the aqueous controls (Figure 4a,b). In *C. crispus*, the uronic acid concentration was 0.8% ± 0.0% (P1), 1.2% ± 0.0% (C1), 1.0% ± 0.0% (C2), and 1.4% ± 0.0% (C3) (Figure 4a). For *C. fragile*, the uronic acid content was 0.23% ± 0.0% and 0.1% ± 0.0% with C1 and P1, respectively (Figure 4b).

#### 2.3.4. Sulfates

The effect of enzyme treatments on the extraction of the sulfated groups is shown in Figure 5a,b. As some red seaweeds are rich in sulfated polysaccharides, the percentage yield of sulfates was higher in *C. crispus*, as compared to *C. fragile*. The percentage of sulfated groups in *C. crispus* enzymatic extracts was 8.4% ± 0.1% (P1), 11.7% ± 0.1% (C1), 8.0 ± 0.1 (C2), and 11.0 ± 0.1 (C3), respectively. For *C. fragile,* no significant differences were observed in the extraction yield of sulfates with the use of P1 (0.5% ± 0.0%) and C1 (0.5% ± 0.0%), as compared to the water extract (0.4% ± 0.1%).

#### 2.3.5. Ash

The percentage yield of ash in the enzymatic hydrolysates was higher than in the water extract (Figure 6a,b). The enzymatic hydrolysates of *C. fragile* had higher ash content than *C. crispus.* For *C. fragile,* the percentage dry matter yield of ash was higher (*p* ≤ 0.05*)* with P1 (35.9% ± 0.3%) than C1 (29.3% ± 0.1%). Moreover, *C. crispus* had a lower yield of ash in the treatments, as well as in their water extract (Figure 6b). For *C. crispus,* the carbohydrases were more effective in extracting ash from the biomass than the proteases (Figure 6b).

### 2.4. Carbohydrate Composition

The monosaccharide profiles of the enzymatic hydrolysates of *C. crispus* and *C. fragile* are shown in Table 2. Extracts were composed of galactose, glucose, and mannose as major sugars and trace quantities of arabinose and xylose were also identified. Xylose was absent in CF extracts and relatively low percentage of arabinose was detected in CC extracts. In CC, the higher percentages of glucose and mannose (46.1% ± 8.6 and 11.7% ± 4.9%, respectively) were detected in the C3 hydrolysate, whereas the percentage of galactose was higher in the control (water blank 83.6% ± 0.4%). In CF, the highest percentage of glucose was found in proteolytic extract (P1, 55.9% ± 0.4%) and galactose, mannose, and arabinose were higher in the control (water blank: 20.06% ± 0.17%, 15.3% ± 0.2%, and 4.5% ± 0.1%, respectively). Besides the detectable monosaccharides, several unknown peaks were observed in CC and CF chromatograms; these were denoted as unknown sugars (mol%). Higher percentages of unknown sugars were detected in CC and CF, the enzymatic hydrolysates (Table 2). Peaks for sugars such as rhamnose, fructose, ribose, glucoheptose, and glucuronic acid were detected at very low levels and, therefore, added to the total for unknown sugars. The polysaccharide content of CC ranged from 40% to 75%), thus indicating that only half of the sugars were being accessed in hydrolysis.

**Table 2:** Monosaccharide composition (mol%) in the enzymatic hydrolysates ^1^.

Treatments	Arabinose	Galactose	Glucose	Mannose	Xylose	Other Sugars
CC (Blank)	0.00	83.63 ± 0.42	1.23 ± 0.19	1.46 ± 0.04	2.57 ± 0.05	10.22 ± 0.31
CC (P1)	0.19 ± 0.01	75.24 ± 1.88	3.13 ± 0.27	1.24 ± 0.71	1.64 ± 0.90	17.95 ± 1.18
CC (C1)	0.07 ± 0.00	73.11 ± 0.39	1.44 ± 0.02	1.72 ± 0.03	2.48 ± 0.06	19.59 ± 0.40
CC (C2)	0.06 ± 0.00	74.87 ± 0.32	1.81 ± 0.23	1.11 ± 0.08	2.45 ± 0.07	18.98 ± 0.09
CC (C3)	3.19 ± 0.44	12.94 ± 2.39	46.05 ± 8.66	11.69 ± 4.93	0.00	28.50 ± 1.97
CF (Blank)	4.49 ± 0.04	20.06 ± 0.17	53.52 ± 0.14	15.30 ± 0.16	0.00	5.85 ± 0.53
CF (P1)	2.48 ± 0.17	10.80 ± 0.77	55.92 ± 0.45	7.25 ± 1.33	0.00	23.21 ± 1.90
CF (C1)	0.12 ± 0.00	78.10 ± 0.37	1.14 ± 0.09	5.45 ± 0.06	0.00	14.27 ± 0.33

^1^ Values are means for each treatment (Trt) group (CC: *Chondrus crispus*; CF: *Codium fragile*; P1: protease 1; C1: carbohydrases 1; C2: carbohydrases 2; C3: carbohydrases 3). Values represent mean ± standard deviation from three independent experiments (*n* = 3).

### 2.5. Antiviral Activity and Cytotoxicity

The enzymatic extracts were tested for their antiviral activity against the *Herpes simplex* virus Type 1 (HSV-1) using Vero cell lines. Additionally, the cytotoxicity of enzymatic extracts to Vero cells was also evaluated. After three days of treatment, no cytotoxicity was observed in extracts of either of the seaweeds tested (Table 3). The enzymatic extracts exhibited significantly higher (*p* < 0.0001) antiviral activity at multiplicity of infection (MOIs) of 0.001 ID_50_/cells (12–45 x acyclovir) and 0.01 ID_50_/cells (3–8 x acyclovir) (Table 3). Maximum inhibition of viral activity was observed in the enzymatic extract of CF (36.5 ± 10.3 μg/mL). In CC*,* the extracts obtained with P1 exhibited effective antiviral effect (77.6 ± 9.6 μg/mL). No anti-HSV-1 activity was observed in CC hydrolysates obtained from enzymatic extraction with C1 and C2 at a multiplicity of infection of 0.01 ID_50_/cells (Table 3). At the same MOI, all the extracts from *C. fragile* showed strong HSV-1 inhibition, with P1 being the most effective (Table 3).

**Table 3:** Evaluation of anti-HSV activity of enzymatic hydrolysate from *Codium fragile* and *Chondrus crispus* by neutral red dye method at MOI = 0.001 and 0.01 ID_50_/cells ^1^.

Treatments ^2^	MOI = 0.001 ID_50_/Cells		MOI = 0.01 ID_50_/Cells	
	CC_50_ (μg/mL)	EC_50_ (μg/mL) ^2^	CC_50_ (μg/mL)	EC_50_ (μg/mL) ^2^
Drug (Acyclovir)	>200	2.9 ± 0.5 ^d^	>200	20.2 ± 8.0 ^c^
CC (Blank)	>200	129.7 ± 0.5 ^a^	>200	>200
CC (P1)	>200	77.6 ± 9.6 ^b^	>200	161.1 ± 4.3 ^a^
CC (C1)	>200	103.3 ± 12.1 ^b^	>200	>200
CC (C2)	>200	126.8 ± 9.0 ^a^	>200	>200
CC (C3)	>200	109.3 ± 9.6 ^b^	>200	178.8 ± 12.3 ^a^
CF (Blank)	>200	52.3 ± 10.7 ^c^	>200	144.3 ± 2.1 ^a^
CF (P1)	>200	36.5 ± 10.3 ^c^	>200	63.2 ± 11.4 ^b^
CF (C1)	>200	41.3 ± 5.6 ^c^	>200	82.8 ± 13.9 ^b^
*P-value*	<0.0001		<0.0001	

^1^ MOI: Multiplicity of infection ID_50_/cells, ratio (Virus titer)/(cell number/mL); cytotoxic concentration (CC_50_): The concentration of a compound required to reduce cell viability by 50% compared to the control. P1: protease 1; C1: carbohydrases 1; C2: carbohydrases 2; C3: carbohydrases 3; effective concentration (EC_50_): the concentration required to achieve 50% protection against virus-induced cytopathy; ^2^ Values represent mean ± standard deviation from three independent experiments (*n* = 12). Values with different superscript letters are significantly different (*p* < 0.05).

## 3. Discussion

In this study, we studied the antiviral activity of enzymatic extracts of the selected, cultivated red seaweed *C. crispus* and green seaweed *C. fragile*. In recent years, the use of enzyme-assisted extraction has gained much attention for its improved recovery of biologically active components from seaweeds [29]. Wang *et al.* (2010) [30] investigated the use of proteases and carbohydrases to improve the yield of polyphenols and other antioxidants from the red seaweed *Palmaria palmata*. In the present study, a proximate analysis was conducted to aid selection of appropriate enzymes for hydrolysis. The proximate composition of *C. crispus* and *C. fragile* showed similar profiles of protein, uronic acid, and neutral sugar content (Table 1), as reported previously in other seaweeds: *Porphyra columbina, Palmaria palmata*, and *Ulva* sp. [31,32]. Fleurence (1999) [33] reported that the crude protein content of dried seaweeds ranged between 11% and 28%, and the present study showed similar levels of protein (Table 1). The ash content of *C. crispus* and *C. fragile* was notably higher than other red and green seaweed species, such as *Sphaerococcus coronopifolius*, *Boergeseniella thuyoides*, *Gracilaria cervicornis*, *Hypnea charoides*, *H. japonica*, and *Ulva lactuca* [23,34,35,36]. This may be due to the differences in species, geographical location, and conditions such as temperature, salinity, and nutrient availability. Previous studies showed that the water extract from *C. fragile* contained about 70% carbohydrates (mainly mannose), and 7%–8% sulfated arabinogalactans [37]. However, in the present study, the neutral sugar percentage in the acid hydrolysates of *C. fragile* was comparatively low (Table 1).

In general, bioactive compounds are extracted with water or organic solvents. However, the extraction efficiency for these methods ranges from 8% to 30% of the algal dry yield [38]. Moreover, bioactive compounds in the seaweed cell matrix are usually present at very low concentrations. Alternative extraction techniques, such as enzymolysis and microwave-assisted extractions, have been employed to improve the yield of extracts [39,40]. Enzyme-assisted extraction (EAE) is an alternative to conventional solvent based methods, due to its high catalytic efficiency, high specificity, and mild reactive conditions [41,42]. Previously, enzyme-assisted extraction has been extensively used to extract bioactives from red and green algae [30,43]. In the present study, carbohydrases (C1) and proteases (P1) were used. However, polysaccharides and gelling agents present in the cell wall of *C. crispus* may have interfered with the extraction process (data not shown). Therefore, the extraction procedure was modified (1:200 mL of water) and two additional carbohydrases (C2 and C3) were used to enhance the extraction of bioactives from *C. crispus*. However, it would have been preferable to use these two carbohydrases in the extraction study of CF for better comparison. The results indicated that enzymes P1, C1, C2, and C3 improved the extraction efficiency (*i.e.*, dry matter yield) two- to threefold as compared to water extraction (Figure 1). Fleurence *et al.* (1995) [44] studied the effect of polysaccharidases (*i.e.*, κ-carrageenase, β-agarase, xylanase, and cellulase) on the extraction of protein from three red seaweeds—*C. crispus*, *Gracilaria verrucosa*, and *Palmaria palmata*. They concluded that *Chondrus crispus/*carrageenase *+* cellulase *and Gracilaria verrucosa/*agarase *+* cellulase combinations resulted in a ten- and three-fold increase in protein extraction, respectively [44]. Similarly, previous studies have reported higher dry matter yields from red seaweeds such as *Solieria chordalis* and *P. palmata* using carbohydrases [2,31]. In red seaweeds including *C. crispus*, the cell wall-bound polysaccharide serves as a substrate for carbohydrases [45]. This correlated to the higher dry matter yield obtained with carbohydrases C1 and C3 from *C. crispus*. Denis *et al.* (2009) [46] used polysaccharidases (Onozuka R-10 cellulase, agarose, and Ultraflo L mixture) to assess metabolite extraction from the red seaweed *Grateloupia turuturu*. The enzymatic treatment resulted in better degradation of *Grateloupia* tissue and a greater release of reducing carbohydrates [46]. Hardouin *et al.* (2014) [2] proposed the use of an enzymatic extraction to increase the yield of antiviral compounds from selected French red, green, and brown seaweeds, *viz.*, *Solieria chordalis*, *Ulva* sp., and *Sargassum muticum*. The use of proteases and carbohydrases resulted in higher yields of protein, neutral sugars, and polyphenols [2]. Similarly, the present study results were in agreement *i.e.*, in comparison to the water extraction, enzymatic extraction resulted in higher recovery of proteins (2–5 x), neutral sugars (2–12 x), uronic acids (2–4 x) and sulfated groups (2–3 x) from the seaweeds *C. fragile* and *C. crispus* (Figure 2, Figure 3, Figure 4, Figure 5 and Figure 6).

Several seaweed polysaccharides, such as carrageenans, sulfate proteoglycans, dextran sulfates, alginate polysaccharide, and sulfated fucans, have been shown to exhibit antiviral activities against human papillomavirus (HPV), influenza A virus (IAV), and human herpes virus HSV-1 and HSV-2 [47,48,49]. Additionally, UVA-photosensitizers from seaweeds have also been identified as virucidal against RNA- and DNA-enveloped viruses. Hudson *et al.* (1999) [50] determined the antiviral activity of extracts from 13 Korean seaweeds. They showed that an organic extract of *C. fragile* was capable of inhibiting three viruses (herpes simplex, HSV; Sindbis, SINV; and polio) [50]. The present experiments confirm the antiviral potential of the enzymatic extracts of *C. crispus* and *C. fragile*. All extracts (enzymatic and water control) had some antiviral activity at MOI = 0.001 ID_50_/cells (Table 3). The fractions obtained with carbohydrases (C1 and C3) and proteases (P1) were the most effective for *C. crispus*. This could be due to the difference in the ability of enzymes to extract sulfates from this red, multiaxial seaweed. Previously it has been shown that the antiviral activity of some seaweed extracts were due to its sulfates that form polysaccharide complexes, similar to the viral cell complex. For example, galactofucan (GFS), a sulfated polysaccharide isolated from the Tasmanian seaweed *Undaria pinnatifida*, inhibited HSV-1 (IC_50_ = 32 μg/mL) and HSV-2 (IC_50_ = 0.5 μg/mL) activity by preventing binding and entry of virus into the host cells [51]. Thus, sulfated polysaccharides from certain seaweeds mimic the cell sulfates and block the entry of viruses into the cells [52]. Additionally, Bourgougnon *et al.* (1993) [21] reported that an aqueous extracts of the red alga *Schizymenia dubyi* extracted a higher sulfate content and was effective in inhibiting HSV-1 replication at EC_50_ = 2.5–80 μg/mL, without a cytotoxic effect. Similarly, in the present study, the *C. crispus* fractions extracted by carbohydrases (C1 and C3) had the higher content of sulfate (Figure 5b, 11.7% ± 0.1% and 11% ± 0.1%, respectively), which could have contributed to the higher antiviral activity observed (Table 3). In *C. fragile*, the fraction obtained from protease (P1) was more conducive for MOI = 0.01 ID_50_/cells (Table 3). This could be due to a higher percentage of glucose (55.92% ± 0.45%) in the protease (P1) fraction of *C. fragile* (Table 2). Previous reports validated the antiviral potential of glucose derivatives including 2-deoxy-d-glucose, uridine 5′-diphosphate glucose, and 1,2,3,4,6-penta-*O*-galloyl-β-d-glucose (PGG) isolated from plant extracts [53,54]. Xiang *et al.* (2011) [55] reported that a glucose derivative, 1,2,4,6-tetra-*O*-galloyl-β-d-glucose (1246TGG), isolated from the traditional Chinese medicinal plant *Phyllanthus emblica* L. (Euphorbiaceae) 1246TGG exhibited antiviral activity against *Herpes simplex* virus Type 1 (HSV-1) and Type 2 (HSV-2) infections by inactivating HSV-1 particles, thus preventing viral attachment and penetration [55]. Further, these sugar molecules have been shown to block the synthesis of the main glycosylated polypeptide of HSV [56]. In CF, the fraction obtained from carbohydrases (C1) also showed significant anti-HSV-1 activity (*p* < 0.0001) Taken together, these results indicate that enzyme-assisted extraction is an efficient method to increase the yield from the selected red and green seaweeds *C. crispus* and *C. fragile*. Additionally, the enzymatic hydrolysates showed enhanced protection to Vero cells against a HSV-1 challenge with no observed cytotoxicity. 

## 4. Materials and Methods

### 4.1. Seaweeds

A cultivated strain of the red alga *C. crispus* (CC) was a kind gift from Acadian Seaplants Limited, Nova Scotia, Canada. The green seaweed *C. fragile* was collected in Nova Scotia, Canada. One kilogram of freshly harvested seaweed was freeze-dried and ground to a powder (0.4 mm, mesh size) using a micro Wiley mill, standard model 3 (Arthur H Thomas Co., Philadelphia, PA, USA). The biomass obtained was stored at −20 °C until used.

### 4.2. Extraction of Seaweeds

#### 4.2.1. Acid Extraction

Acid hydrolysis was performed using the methods described by Defer *et al.* (2009) [57], with minor modifications. Two experiments were performed, using 10 mg of ground seaweed. The hydrolyzed materials were extracted in 5 mL of hydrochloric acid (1N HCL) in a heat block (100 °C) for 2 or 5 h. Composition of the hydrolyzed extracts was analyzed after neutralizing with sodium hydroxide (5 mL NaOH). 

#### 4.2.2. Enzyme Extraction

**Scheme 1:**
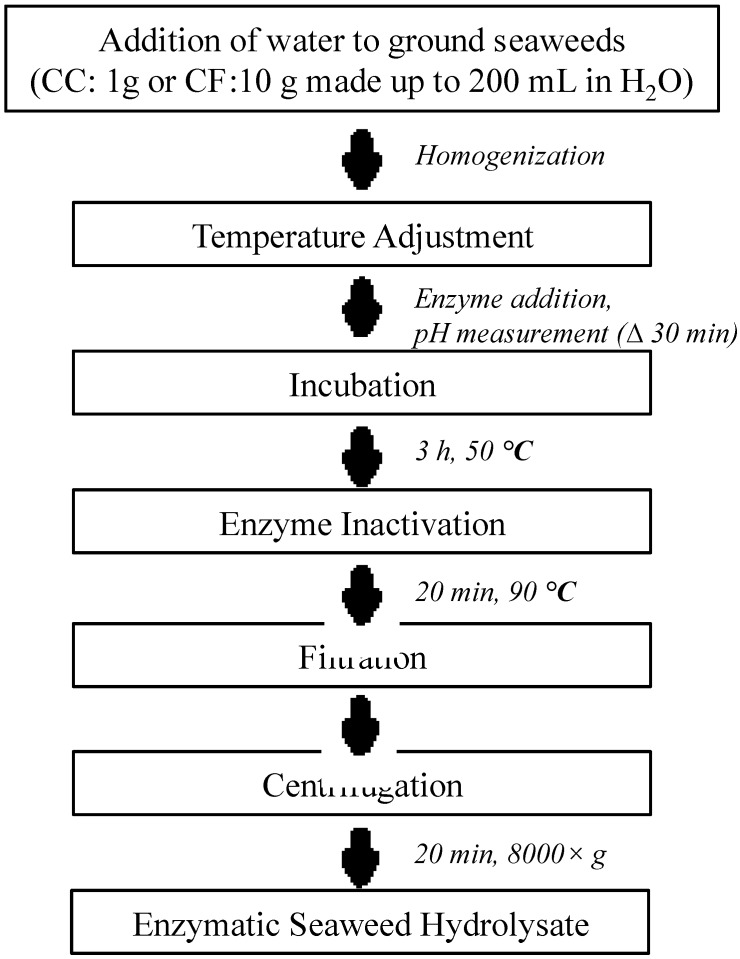
Schematic representation of seaweed extraction process.

Four commercial enzymes from Novozyme—cellulase (C1), β- glucanase (C2), ultaflo (C3), and proteases (Neutrase)—were used for the extractions. Hydrolysis was performed as described by Hardouin *et al.* (2014) [2], with minor modifications. The extraction procedure is summarized in Scheme 1. Enzyme-assisted extraction was performed by adding ground seaweed powder (1 and 10 g of CC and CF, respectively) to 200 mL of distilled water. Enzymes were added at a concentration of 0.5% and were placed in a 50 °C water bath for 3 h, after which the enzyme was denatured by raising the temperature to 90 °C for 15 min. The extraction process for *C. crispus* was modified (1 g of CC in 200 mL of water) with two additional enzymes (C2 and C3) to overcome the interference of gelling agents present in its cell wall. The hydrolysates were centrifuged at 8000× *g* for 15 min at 20 °C to remove the undigested residue. The resulting supernatant was filtered, freeze-dried, weighed, and stored at −20 °C for further experiments.

#### 4.2.3. Water Extraction

The water extract served as a control in all experiments. Water extract was produced by adding 1 and 10 g of freeze dried seaweed powder (CC and CF, respectively) to 200 mL of distilled water and extracted at 50 °C for 3 h.

### 4.3. Analysis of Chemical Composition of Extracts of Seaweed

Neutral sugars in the extract were determined by the phenol-sulphuric acid colorimetric method, as described by Dubois (1956) [58] using anhydrous d-glucose (0–100 μg/mL) as the standard. The uronic acid content was quantified using the meta-hydroxy-di-phenyl (MHDP) method [59] with gluconic acid as the standard. Following acid hydrolysis of the soluble polysaccharides, the free sulfates were measured by the Azure A method [60]. For this estimation, sulfated dextran (17%) (0–100 μg/mL) was used as the standard. The bicinchinonic acid colorimetric method (BCA) [61] with a Micro BC assay kit (cat. UP75860C, INTERCHIM) was used to measure the protein content. For the protein estimation, Bovine Serum Albumin (0–100 μg/mL) was used as the standard. Total ash was determined by incinerating the ground seaweeds at 550 °C for 16 h, followed by 900 °C for 2 h. 

### 4.4. Analysis of Carbohydrates in the Seaweed Hydrolysates

High-performance anion-exchange chromatography (HPAEC) (Dionex, Sunnyvale, CA, USA) was used to determine the composition of carbohydrates in the extracts. The extracts were hydrolyzed with hydrochloric acid (1M) for 48 h at 100 °C. The hydrolysates obtained were neutralized with sodium hydroxide (1 M) and then filtered for analysis. An analytical column (CarboPac PA1, 4 × 250 mm) was used and the elution (1 mL/min and 110 bars) was carried out under alkaline conditions. The eluents used were: Milli-Q water (A), 100 mM NaOH (B), and 100 mM NaOH + 1M NaOAc (C). The injection volume was set to 2 μL and following elution program was used: 20 min isocratic (A/B 80:20), 5 min linear gradient (from C 100%), followed by 5 min isocratic conditions (C, 100%) and re-equilibrium for 20 min at isocratic conditions (A/B, 80:20). Carbohydrates were detected by pulsed amperometry with a detector composed of a silver standard electrode and a gold working electrode. Commercially available reference compounds (e.g., ribose, glucose, rhamnose, galactose, arabinose, xylose, mannose, fructose, guloheptose, and glucuronic acid) were used as internal standards. The chromatograms were analyzed using Chromeleon^®^ software (Dionex, Sunnyvale, CA, USA). Carbohydrates were identified and quantified by using calibration curves of internal standards (50 µM).

### 4.5. Determination of Antiviral Activity

#### 4.5.1. Cells and Viruses

The African green monkey kidney cell line (Vero, ATCC CCL-81) was used for *Herpes simplex* Type 1 (HSV-1) replication. The Eagle’s minimum essential medium (MEM, Eurobio, Courtaboeuf, France), supplemented with 8% fetal calf serum (FCS, Eurobio Courtaboeuf, France) and 1% antibiotics PCS (10,000 IU/mL penicillin, 25,000 IU/mL colimycin, 10 mg/mL streptomycin, Sigma, Saint-Quentin Fallavier, France) was used as the culture medium. The virus stock of HSV-1, a wild-type strain 17 (sensitive to acyclovir), was a kind gift from Agut (Laboratoire de Dynamique, Paris, France).

#### 4.5.2. Cytotoxicity of Seaweed Hydrolysates

The cytotoxicity of the seaweed extracts was tested on the Vero cell/HSV-1. Briefly, the cell suspensions (3.5 × 10^5^ Vero cells/mL) were incubated with the seaweed hydrolysates (1–200 μg/mL, 4 wells per concentration). Each dilution (50 μL) was made in Eagle’s MEM with 8% FCS, dispensed in 96-well (Nunclon, Intermed, Saint-Quentin Fallavier, France) micro-test III tissue culture plates, and incubated at 37 °C for 72 h with an atmosphere of 5% CO_2_. The culture medium, without the extract, served as the control. Cells were observed daily under a phase-contrast microscope to establish the minimum concentration of hydrolysate required to induce alternations in cell morphology (e.g., swelling, shrinkage, granularity, and floating). Cytotoxicity, based on cell viability, was determined using the neutral red dye assay [62]. Absorbance was read using a multi-plate spectrophotometer (Packard Spectra Count™) at 540 nm. The 50% cytotoxic concentration (CC_50_) of a given extract (expressed as percentage) was defined as the concentration that reduced the absorbance of treated cells to 50% of that of the untreated control. This was calculated as [(ODc) C − (ODc) MOCK/(ODc) C] × 100, where (ODc)C and (ODc) MOCK were the OD (optical density) values of the untreated cells and treated cells, respectively [63]. 

#### 4.5.3. Antiviral Activity of Seaweed Hydrolysates

The selected seaweed hydrolysates (1–200 μg/mL, 50 μL) were diluted in Eagle’s MEM containing 8% FCS and incubated (37 °C, 5% CO_2_) with cellular suspensions (3.5 × 10^5^ Vero cells/mL, 100 μL) in 96-well plates. The cells were infected with a virus cell suspension (50 μL) at two MOIs of 0.01 and 0.001 ID_50_/cells. MEM medium with 8% FCS (50 μL) was added to the mock treatments. Following incubation (72 h), the antiviral activity was evaluated by the neutral dye method [62]. The anti-herpetic compound Acyclovir (9-(2-hydroxyethoxymethyl guanine) (Merck, Whitehouse Station, NJ, USA), a commercial HSV-1 inhibitor, served as the positive control. Results of antiviral activity were expressed as effective antiviral concentration (EC_50_). The percentage of cell protection against the viral infection (%P) was calculated using the following formula:

[((ODt) virus − (ODc) virus)/((ODc) MOCK − (ODc) virus)] × 100

where (ODt) virus was the OD (optical density) of the test sample, (ODc) virus was the OD of the virus-infected control (no samples), and (ODc) MOCK was the OD of the mock-infected control. The concentration of seaweed hydrolysate, which provided 50% protection to virus-infected cells, was determined from a dose-response curve using linear regression [62,63].

### 4.6. Statistical Analysis

The experiment was set up in a completely randomized design. In quantification assays, linear regression analysis was carried out for standards using a scatter plot (Microsoft excel^®^, 2012). Results are expressed as mean ± standard deviation (SD). Each experiment was repeated three times and was conducted in triplicate. The data were analyzed using ANOVA, with a *P* value < 0.05 using the Proc. mixed procedure, of the SAS Institute, Inc. software version 9.3 (SAS Institute, Inc., Cary, NC, USA). When significant effects of treatments were found, multiple means comparison was carried out using Tukey’s analysis with a 95% confidence interval and α = 0.05 (α—level of significance) to differentiate treatment means.

## 5. Conclusions

The green and red seaweeds used in this study, *C. fragile* and *C. crispus*, were subject to enzymatic hydrolysis. The extracts produced were tested for their antiviral activity. *C. crispus* was characterized by higher levels of protein and sulfate and *C. fragile* had a higher amount of neutral sugar and ash. The commercial enzymes tested showed higher yields of bioactive materials in both seaweeds. Since enzyme extraction is solvent-free, the hydrolysates obtained were of food grade and could be easily utilized in the cosmetics and food industries. Furthermore, extracts obtained from enzyme-assisted hydrolysis exhibited antiviral activity with EC_50_ values in the range of 77.6–129.8 μg/mL for CC and 36–52 μg/mL for CO, at a MOI of 0.001 ID_50_/cells without cytotoxity. Additionally, even at higher MOI (0.01 ID_50_/cells), the enzymatic extracts from proteases (P1) and carbohydrases (C1 and C3) revealed significant antiviral activities. Ion exchange chromatography of extracts identified the presence of five simple sugars. The percentage of glucose was significantly higher in the enzymatic extract (P1) of both seaweeds tested. Interestingly, derivatives of glucose have been reported as anti-HSV compounds. In this regard, the tested seaweeds could be used in therapeutics to generate potential new antiviral drugs for humans. In summary, enzymatically produced extracts of *C. fragile* and *C. crispus* exhibited promising anti-HSV activity that could be used for novel, functional applications in pharmaceutical, nutraceutical, and functional food applications. 
